# Testing the predictions of sex allocation hypotheses in dimorphic, cooperatively breeding riflemen

**DOI:** 10.1002/ece3.3934

**Published:** 2018-03-06

**Authors:** Nyil Khwaja, Stephanie A. J. Preston, James V. Briskie, Ben J. Hatchwell

**Affiliations:** ^1^ Department of Animal & Plant Sciences University of Sheffield Sheffield UK; ^2^ School of Biological Sciences University of Canterbury Christchurch New Zealand

**Keywords:** Acanthisittidae, cooperative breeding, parental care, provisioning rate, repayment hypothesis, rifleman, sex ratio, sexual dimorphism

## Abstract

Evolutionary theory predicts that parents should invest equally in the two sexes. If one sex is more costly, a production bias is predicted in favour of the other. Two well‐studied causes of differential costs are size dimorphism, in which the larger sex should be more costly, and sex‐biased helping in cooperative breeders, in which the more helpful sex should be less costly because future helping “repays” some of its parents’ investment. We studied a bird species in which both processes should favor production of males. Female riflemen *Acanthisitta chloris* are larger than males, and we documented greater provisioning effort in more female‐biased broods indicating they are likely costlier to raise. Riflemen are also cooperative breeders, and males provide more help than females. Contrary to expectations, we observed no male bias in brood sex ratios, which did not differ significantly from parity. We tested whether the lack of a population‐wide pattern was a result of facultative sex allocation by individual females, but this hypothesis was not supported either. Our results show an absence of adaptive patterns despite a clear directional hypothesis derived from theory. This appears to be associated with a suboptimal female‐biased investment ratio. We conclude that predictions of adaptive sex allocation may falter because of mechanistic constraint, unrecognized costs and benefits, or weak selection.

## 1 INTRODUCTION

Theory predicts that parents should invest equally in sons and daughters (Fisher, 1930). All other factors being equal, this may be achieved by random sex allocation at a 1:1 ratio and a fixed level of investment per parent. However, a number of factors are predicted to promote production biases toward a particular sex, especially where the sexes differ in behavior, ecology or morphology. In particular, where one sex is costlier to produce, the evolutionarily stable sex ratio is predicted to be biased against this costly sex, at a point where the fitness benefits it gains from rarity balance the extra cost of its production (Hamilton, 1967). For the same reason, if producing one sex confers a benefit to breeders, the optimal sex ratio is predicted to be biased toward it (Emlen, Emlen, & Levin, 1986).

In sexually size‐dimorphic species, it is likely (and generally assumed) that the larger sex is costlier to produce (e.g., Benito & González‐Solís, 2007; Stamps, 1990). In some species, this assumption has been questioned because studies have been unable to demonstrate differential costs (e.g., Laaksonen et al., 2004). Therefore, providing evidence to support this assumption is an important first step in making evolutionary predictions about optimal sex ratios (Magrath, Van Lieshout, Pen, Visser, & Komdeur, 2007). In bird species where parents’ provisioning rate is a valid measure of their parental investment, the larger sex should require greater provisioning effort if it is genuinely costlier to produce (Nishiumi, Yamagishi, Maekawa, & Shimoda, 1996).

In cooperative breeders, parents are typically assisted in reproduction by nonbreeding helpers. Where cooperation is kin‐based, helpers are close relatives (often previous offspring) that usually enhance breeders’ reproductive success (Koenig & Dickinson, 2016; Riehl, 2013). Helping is usually sex‐biased (Komdeur, 2004). Emlen et al.'s (1986) “repayment hypothesis” showed theoretically that the sex that provides more help is effectively less costly to produce, because of the greater probability of it repaying its parents’ investment through future help, which in turn increases the parents’ reproductive success. The hypothesis predicts the production of brood sex ratios biased toward the more helpful sex at the population level, and this prediction has been substantiated in some empirical studies (e.g., Clarke et al., 2002; Woxvold & Magrath, 2008), although not in several others (e.g., Koenig & Walters, 1999; Nam, Meade, & Hatchwell, 2011).

Even when sexes differ in their production cost, population brood sex ratios may not be biased if individual breeders can manipulate the sex of their offspring according to adaptive cues (Frank, 1995; Trivers & Willard, 1973). For example, breeding female Seychelles warblers *Acrocephalus sechellensis* show sophisticated control of their broods’ sex ratios. Female offspring are philopatric and more likely to help in this species, but production is biased toward females only on territories of sufficient quality to support extra group members, and especially when breeders do not already have helpers (Komdeur, Daan, Tinbergen, & Mateman, 1997). Similarly, in western bluebirds *Sialia mexicana*, facultative sex determination by breeding females is dependent on resource availability (Dickinson, 2004). Although in these cases, sex allocation appears to have been finely tuned by natural selection, population sex ratios are not biased toward the apparently cheaper, more helpful sex (Koenig & Dickinson, 1996; Komdeur et al., 1997). These studies have also provided support for the “local resource competition” hypothesis, in which philopatric offspring competing for their parents’ resources incur a cost that may mitigate the benefits they provide by helping (Clark, 1978). However, such facultative sex manipulation is relatively rare (Khwaja, Hatchwell, Freckleton, & Green, 2017), and theoretical work suggests that even small costs are likely to outweigh any adaptive benefits of sex ratio control (Pen, Weissing, & Daan, 1999).

An additional problem in understanding the adaptation of offspring sex ratios is that these various selection pressures on sex allocation may confound each other, and make theoretical predictions of biased brood sex ratios problematic. For example, in most cooperative breeders the more helpful sex is also more philopatric and therefore likely to compete with its parents for resources (Clark, 1978). Likewise, male birds are generally more helpful, but they also tend to be larger than females, so any repayment benefits may be offset by higher production costs (Komdeur, 2004).

Riflemen *Acanthisitta chloris* are cooperatively breeding birds, which are unusual (perhaps unique) in that the three discussed selection pressures unambiguously suggest that offspring sex ratios should be biased in one direction: toward males. Firstly, female riflemen are considerably larger than males even as nestlings, and hence are likely to be costlier to produce (Sherley, 1993). Secondly, helping is male‐biased (72% of 32 adult helpers observed during our study were male). Adult helpers in this species are associated with enhanced breeding productivity (Preston, Briskie, & Hatchwell, 2016), and most are previous offspring of the breeders they help (Preston, Briskie, Burke, & Hatchwell, 2013), suggesting that sons are more likely than daughters to repay a portion of their production cost. Thirdly, males and females do not differ significantly in their natal dispersal distances, and adult helpers are established on their own territories from which they “commute” to the territory of their recipient brood (Preston, 2012). Therefore, it is unlikely that the value of repayment is diminished by local resource competition. The alignment of these factors means that riflemen provide a rare opportunity to test a strong directional prediction of biased offspring sex ratios in a natural population.

In light of this strong prediction, the results of a previous study by Sherley (1993) that showed unbiased sex ratios in nestling riflemen are especially surprising. In this paper, we test the assumption that females are costlier to raise than males by observing patterns of provisioning, and report brood sex ratios of riflemen over six breeding seasons, including using molecular sexing of nestlings that died early to approximate the primary sex ratio. We then test the hypothesis that an unbiased population sex ratio masks biases at the individual level, in which females facultatively produce broods with sex ratios that are adaptive to their context. For example, breeders without helpers may benefit more by producing helpful males, or those in better condition may be better able to produce costlier females (Emlen et al., 1986; Trivers & Willard, 1973). Finally, we discuss the implications of our results for understanding variation in sex allocation within and between species.

## 2 MATERIALS AND METHODS

### 2.1 Data collection

We studied a nestbox population of riflemen at Kowhai Bush (173°37′E, 42°23′S), near Kaikoura on New Zealand's South Island, during six breeding seasons (September–January) from 2008 to 2011 and 2012 to 2015. The population ranged between 6 and 23 breeding pairs during this time. Each individual in the population was given a unique combination of two color rings and a metal Department of Conservation AP ring for identification, either as a 15‐day‐old nestling or as an adult or juvenile caught by mist‐netting near to known nests.

Active nests were identified by weekly checking of all nestboxes on the study site for the presence of nests, and daily checks of those boxes containing nests. All nests were followed from clutch initiation through to fledging. Females produce clutches of two to five eggs, laid at intervals of 2 days, which are incubated by both sexes for 18 to 21 days. Rifleman pairs made a maximum of two successful reproductive attempts in a breeding season. Broods of pairs that had already fledged offspring in a given breeding season were considered “second broods”; all others were considered first broods. When possible, each nest was filmed using a digital camcorder every 3 days after hatching, starting at day 3, where hatching is defined as day 0 (nestlings typically fledged around day 24). Each recording started with a 15‐min acclimatization period for which footage was discarded, with data then collected from the following hour. Recording start time varied between 0700 and 1700 NZST. Carers were never caught on the days their nests were filmed. Data were transcribed from these videos to obtain provisioning rates for each carer (Khwaja, Preston, et al., 2017).

After nests were filmed on day 15, each nestling was temporarily removed from the nest to be weighed, measured, ringed, sexed, and have samples taken of blood (for genetic analysis) and preen wax (for chemical analysis, used in a different study). At least one nestling was left in each nest at all times so that adults did not return to an empty nest, which may stimulate abandonment. Rifleman nestlings are sexually dimorphic and can be sexed in the hand by day 15, females being >10% larger with differently colored plumage (Sherley, 1993). The reliability of morphological differences to sex birds was confirmed using the Z043B microsatellite marker (Dawson, Dos Remedios, & Horsburgh, 2016); this marker was also used to sex nestlings that died prior to day 15, from which tissue samples were collected. These nestlings could be collected from the nests as the nestboxes at the site are generally inaccessible to predators (Briskie, Shorey, & Massaro, 2014). We obtained Queller‐Goodnight pairwise relatedness estimates between members of a breeding pair using DNA extracted from blood or tissue, amplified at 16 additional microsatellite loci (Preston, Dawson, Horsburgh, & Hatchwell, 2013; Table A1), using the program SpAGeDi (Hardy & Vekemans 2002).

### 2.2 Data analysis

Statistical analyses were carried out using R 2.12.0 (R Development Core Team, Vienna, Austria). We used generalized linear mixed‐effects models in the lme4 package (Bates, Maeschler, Bolker, & Walker, 2015) to investigate whether brood sex ratio influenced investment by carers, and whether any potential adaptive cues were associated with adaptive sex allocation in broods. For the former, we modeled carer visit rates as a Poisson‐distributed response variable, with brood sex ratio (numeric: proportion of males) as a fixed predictor along with potential confounds: number of nestlings (numeric), nestling age (numeric: in days), carer status (factor: parent or helper), sex of carer (factor), brood order (factor: first or second brood), date (numeric: number of days from 1st September), time of day (numeric: number of hours from 0700 hr), and season (year of study). We initially included the interactions between brood sex ratio and both carer status and sex, but removed these as they received no statistical support. Individual identity and territory were fitted as random effects. Visit rate is an appropriate measure of investment by provisioning riflemen, as it does not trade off against load size as in some other systems (Khwaja, Preston, et al., 2017).

Brood sex ratio was modeled using a binomial error structure with a two‐column response variable: number of males and number of females. This allowed the proportion of males to be examined with appropriate weight given to the total brood size (Crawley, 2007). We fitted population density (numeric: number of pairs breeding within 200 m of nest), brood order (factor: first or second brood), whether a brood was helped (factor), brood size (numeric), season (factor: 2008–2009, 2009–2010, etc.), and pairwise relatedness estimate between male and female parents (numeric) as explanatory fixed predictors. Pair identity nested within mother identity was fitted as a random effect. Father identity was not included as females are the heterogametic sex in birds, meaning males are unlikely to contribute directly to sex allocation (Rutkowska & Badyaev, 2008); fitting pair identity accounted for the potential effect of partner on female allocation decisions. Preston, Briskie, et al. (2013) detected no extra‐pair paternity in this population, so we assumed that social fathers sired all offspring in a brood. We used the intercept term in this model to determine whether brood sex ratios differed significantly from parity. To consider the evidence for facultative sex allocation, we assessed the significance of explanatory variables in this model as potential adaptive cues. We also examined whether allocation patterns at the nest level differed from those at the population level: for each size of brood (one, two, three, four, and five) we compared the frequency at which each proportion of males was observed with that expected if males were produced with a uniform probability at each nest. We used exact multinomial tests for these comparisons, except for broods with one nestling, where we used the exact binomial test as there were only two possible outcomes (no males or one male).

Based on our results for the body size of each sex and the effect of brood sex ratio on provisioning rate, we estimated the percentage cost difference in producing male and female offspring. Based on this, we predicted an “adaptive” brood sex ratio, assuming as in Fisher's (1930) principle that this percentage difference generates selection pressure to produce sex ratios that are biased to the same degree. We compared observed sex ratios to this adaptive prediction using exact binomial tests.

## 3 RESULTS

### 3.1 Size dimorphism

**Figure 1:**
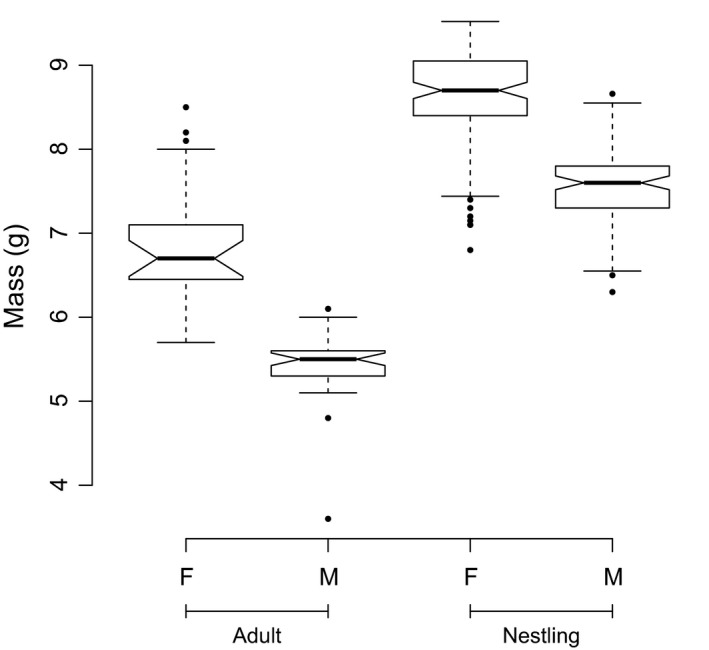
Differences in mass between female and male riflemen captured as adults (23 females and 40 males; *t *=* *8.94, *df* = 29, *p* < .001) and weighed as 15‐day‐old nestlings (111 females and 93 males; *t* = 14.96, *df* = 200, *p* < .001). The analysis was restricted to the 2012 to 2015 dataset to avoid uncontrolled observer effects

Adult females were larger than males, and this dimorphism was also apparent when nestlings were weighed at day 15 (Figure 1). On average, females were 27% heavier than males as adults, and 14% heavier as nestlings.

### 3.2 Investment in relation to brood sex ratio

**Figure 2:**
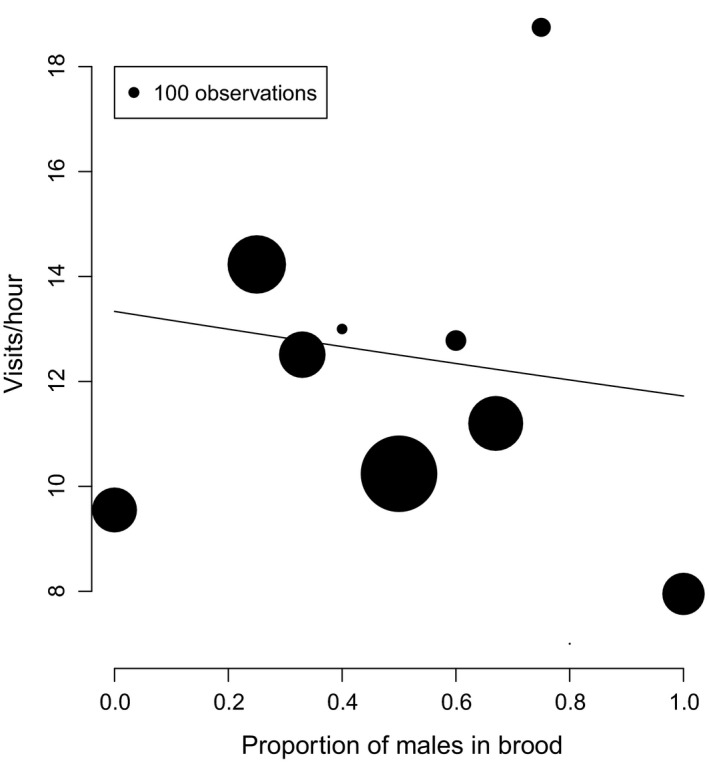
The effect of brood sex ratio on carer visit rates in riflemen. Points show mean number of visits per carer recorded in an hour, summarized for each observed proportion of males and scaled by sample size. The line is fitted from a generalized linear mixed‐effects model with brood size, nestling age, date, and time set to their mean values, status set to “breeder” and sex to “female” (breeding females provision intermediately between breeding males and helpers; there are no significant differences in the slope of the relationship depending on carer status or sex)

We analyzed provisioning data from 1,124 provisioning hours of 122 different carers feeding 97 broods (mean carer visit rate = 11.57 ± 0.25 *SE* visits per hour). Carers showed a significant response to brood sex ratio in their provisioning investment. Broods with a higher proportion of males received fewer provisioning visits during the nestling period (GLMM: β = −0.12 ± 0.05 *SE*,* z *=* *−2.42, *p* = .016; Figure 2; Table A2). Back‐transformed to the dimensions of visits per hour, the estimated effect equates to a difference of 1.61 visits per carer between all‐female and all‐male broods, representing 14% of mean provisioning rate (Figure 2).

### 3.3 Brood sex allocation

We determined the sex of 289 nestlings in 93 rifleman broods over six breeding seasons. We were unable to assign sex to nine nestlings (3%) from seven different broods, all of which died early in the nestling period. Of the 289 successfully sexed nestlings, 134 (46%) were male and 155 (54%) were female. The mean proportion of males across broods was 0.47 ± 0.03 *SE* (*n *=* *93), which does not represent a significant deviance from parity (see intercept term in Table 1). We estimated an adaptive production sex ratio to be 53% male, based on females being 14% costlier as suggested by observed differences in body mass and provisioning effort. The observed numbers of males and females produced differed significantly from this “adaptive” ratio (exact binomial test: *p* = .025). In total, 37 sexed nestlings failed to survive to fledging, of which 16 were male and 21 female. Thus, of 252 fledglings, 118 (47%) were male and 134 (53%) were female, a sex ratio that did not differ significantly from parity or from that for all nestlings (binomial and χ^2^ tests: *p* > .1), and which differed marginally nonsignificantly from that predicted under the adaptive hypothesis (exact binomial test: *p* = .051).

**Table 1:** Effect estimates on the logit scale from potential predictors of brood sex ratios in riflemen, modeled as fixed effects in a binomially‐distributed generalized linear mixed‐effects model, with the proportion of male offspring in a brood as the response variable (*n* = 80 broods). Pair identity (variance component < 0.01) nested within female identity (variance component < 0.01) was included as a random effect along with breeding season (variance component < 0.01). Second brood and helped are categorical predictors with first broods and unhelped nests as respective reference categories. All results were qualitatively equivalent when 13 more broods were included without estimates of mother–father relatedness (Table A3); when nine unsexed nestlings, which we omitted from the model presented, were treated as all male (Table A4) or all female (Table A5), and when number of helpers (0–4) was included as a covariate instead of a categorical “helped” variable (Table A6)

Predictor	β ± *SE*	*z*	*p*
*Intercept*	−0.58 ± 0.58	−1.01	.314
Density (no. pairs within 200 m)	0.10 ± 0.06	1.76	.078
Second brood	0.04 ± 0.38	0.11	.911
Helped	0.21 ± 0.29	0.72	.475
Brood size	<0.01	0.03	.973
Mother–father relatedness	0.26 ± 0.60	0.43	.666

We found no evidence for individual‐level sex ratio manipulation by breeding females. Firstly, there was no indication of a departure from the population sex ratio at the level of individual broods (exact binomial and multinomial tests: *p* > .2 for all brood sizes). Secondly, none of the potential adaptive cues we tested had a significant effect on brood sex ratios, although greater local population densities had a marginal positive effect on the proportion of males produced (Table 1).

## 4 DISCUSSION

We found that female nestling riflemen were significantly heavier than male nestlings and that rifleman carers provisioned broods more frequently when they were more female‐biased. Furthermore, helping is male‐biased in this species. However, despite these indications that daughters are costlier to produce than sons, we found no evidence that sex allocation was either skewed toward males or responsive to any cues regarding the future value of offspring.

Riflemen show pronounced sexual dimorphism, with female adults 27% larger than males. This is unusual among birds, where males are more commonly larger than females, and the majority of species are closer to monomorphism (Székely, Lislevand, & Figuerola, 2007). Sherley (1985) suggested that female riflemen are unable to evolve to a more optimal smaller size because of the constraint of egg production, although this cannot explain why other small species do not also show similar levels of reversed sexual dimorphism. Size dimorphism and sexual dichromatism could also, or alternatively, represent adaptations to different foraging microenvironments (Hunt & McLean, 1993). Whatever the reason for the pattern in riflemen, size dimorphism carries clear implications for the cost of producing each sex.

Where one sex is costlier to rear than the other, the evolutionarily stable sex ratio should be biased against it, as the additional costs mitigate the enhanced reproductive success enjoyed by the rarer sex (Fisher, 1930; Hamilton, 1967). It is generally assumed that the larger sex is costlier to produce in dimorphic species and that this influences optimum sex ratios and sex allocation (Benito & González‐Solís, 2007). However, demonstrating this empirically can be problematic (Magrath et al., 2007). Here, we found elevated provisioning rates at female‐biased broods. This does not cover the entire period of investment, as parents also produce and incubate eggs and feed offspring after fledging. Nevertheless, the magnitude of the difference between provisioning rates at all‐male and all‐female broods is remarkably consistent with the difference in body mass between the sexes at 15 days old (both 14%). We suggest the likeliest explanation is that female riflemen are costlier to raise than males, as the difference in provisioning cost is as expected from their difference in size. Sherley (1993) reported that female riflemen in all‐female broods weighed less than those in mixed broods, suggesting that the costs of parental care in completely female broods may exceed the ability of parents to provision adequately. Taken together these findings suggest that elevated provisioning rates at female‐biased broods are genuinely costly to carers, because they are unable or unwilling to adjust sufficiently for female offspring to grow to optimal size. As with male brown songlarks *Cinclorhamphus cruralis* (Magrath et al., 2007), multiple lines of evidence, therefore, support the case that female offspring are costlier to raise in riflemen. Our analysis suggests that more frequent food delivery is a key mechanism through which this cost is realized. An absence of significant interactions indicates that it is shared by all carers during provisioning.

In addition to the differential production costs of males and females, recent genetic analysis has demonstrated that riflemen are kin‐based cooperative breeders, with most helpers being male offspring of the breeding pair (Preston, Briskie, et al., 2013). These selection pressures of male‐biased helping and costly female production, combined with a lack of local resource competition, generates a predicted bias in offspring sex ratio that is clearer than in any other avian cooperative breeder we are aware of. Thus, Sherley's (1993) earlier observation of even brood sex ratios in riflemen was intuitively surprising. Nevertheless, over six seasons of study, our results were remarkably similar. The population sex ratio was not significantly different from parity, but did differ significantly from an adaptive hypothesis that optimal offspring sex ratios would be 53% male owing to their estimated cheaper cost of production (note that this hypothesis is conservative as it only accounts for estimated cost differences in production and not the effect of repayment; we therefore likely underestimate the difference between our results and adaptive predictions). Taken alone, our results appear to show strong support for the contention that production sex ratios are constrained at parity and not subject to selection, although it is noteworthy that pooling them with Sherley's (1993) yields a *female* majority of 53% (*n *=* *768) that is almost statistically significant (binomial test: *p* = .052). Adaptationist explanations are difficult to conceive. Adaptively biased sex ratios may be generated by differential nestling mortality, rather than skewed production (Komdeur & Pen, 2004), but this is not indicated by our data: more females than males died in the nest, but this difference was not significant and did not cause a male bias at fledging (more females fledged than males). Although the mortality regime for rifleman nests at Kowhai Bush is not natural, because nestboxes afford almost full protection from predators (Briskie et al., 2014), this is unlikely to mask female‐biased mortality given that nest predation generally results in the total loss of all nestlings in a brood (also, most “natural” mortality is due to exotic mammalian predators, which have been sympatric with riflemen only since human settlement of New Zealand).

The other feasible adaptive explanation would be that breeding females adjust the sex of their offspring according to their context, but this explanation also was not supported by our results. We tested the influence of five potential correlates of brood sex ratio, each of which (or a related trait) has been demonstrated to influence sex determination in other species (Griffin, Sheldon, & West, 2005; Howe, 1977; Komdeur et al., 1997; Sardell & DuVal, 2014; Woxvold & Magrath, 2008). None showed a convincing effect. Although this may be attributable to low statistical power, the overall distribution of brood sex ratios barely deviated from expectations based on the population mean, suggesting no variation in sex allocation strategies by different females and therefore no evidence for facultative sex ratio manipulation.

Our results are more consistent with the perspective that sex ratio biases are constrained and/or that hypothesized patterns of biased sex allocation are not valid, at least in this species. Biased production of males and females is apparently not mechanistically prevented in birds generally, as several robust studies have documented significant deviations from parity in production at the population level (e.g., Cockburn & Double, 2008; Koenig, Stanback, Haydock, & Kraaijeveld‐Smit, 2001). Nevertheless, it is possible that mechanisms of biased production are taxon‐specific (Rutkowska & Badyaev, 2008), in which case brood sex ratio may not respond to selection in some species. Similarly, there are some striking examples of facultative sex determination in birds (e.g., Komdeur et al., 1997; Sheldon, Andersson, Griffith, Ornborg, & Sendecka, 1999), but also many negative results (Khwaja, Hatchwell, et al., 2017; West & Sheldon, 2002). If the mechanistic basis for sex allocation bias is either constrained, costly to implement, or costly to evolve, patterns of sex determination may appear suboptimal (Pen et al., 1999).

Alternatively, predictions of optimal brood sex ratios may falter because of the confounding effect of “cryptic” fitness benefits, which require long‐term study to detect (Koenig & Walters, 1999). For example, a higher initial cost of producing one sex may be offset if it reaches postfledging independence sooner than the other, and a “repaid” cost of producing the more helpful sex may be offset if it suffers lower reproductive success. Differences in lifetime fitness between the sexes, for example owing to sex‐biased recruitment or adult survival, could make the fitter sex more beneficial to produce in a way that counteracts (or exaggerates) selection driven by repayment or differential production costs. We have insufficient data to parameterise lifetime fitness accurately in riflemen. However, it is notable that in studies with sufficient long‐term data to incorporate into adaptive predictions of brood sex ratios, these have still predicted sex ratios biased to the helping sex, and as here have been significantly different to those that were observed (Koenig & Walters, 1999; Koenig et al., 2001).

Another possibility is that selection on sex ratios may be weak in many cases. In kin‐based cooperative breeders, help is often allocated preferentially or exclusively to close relatives (Cornwallis, West, & Griffin, 2009). Where this is the case, potential repayment depends not only on the sex of offspring produced but also on the survival of each of the breeding pair. Where this is low it is likely to weaken the adaptive value of overproducing the helpful sex. Furthermore, in species such as the rifleman or long‐tailed tit *Aegithalos caudatus* where helpers are often failed breeders that redirect their care to help kin, the production of potential breeders may take priority over the production of potential helpers (Nam et al., 2011).

In conclusion, our results indicate a pattern of sex allocation in riflemen that sex ratio theory predicts should be disadvantageous (Fisher, 1930). This paper joins an equivocal literature on vertebrate sex allocation, in which extraordinary examples of adaptation sit alongside studies such as this in which plausible fitness benefits are apparently not realized by breeders (Cockburn & Double, 2008; Khwaja, Hatchwell, et al., 2017; West & Sheldon, 2002). In particular, we show that the strength of an intuitive prediction is not necessarily reflected in a sex ratio bias at production. Whether this is because of mechanistic constraint, weak selection, or something else, remains to be determined. Advances in our understanding of proximate mechanisms of sex ratio bias are likely to make a valuable contribution to this field.

## CONFLICT OF INTEREST

None declared.

## AUTHOR CONTRIBUTIONS

N.K., B.J.H., and J.V.B. conceived and designed the study. S.A.J.P. and N.K. collected field data and conducted laboratory work. N.K. and B.J.H. carried out the analyses. N.K. wrote the first draft of the manuscript, to which all coauthors contributed revisions.

## APPENDIX 1

### 1.1

**Table A1:** Markers used to genotype 89 breeding riflemen (46 males and 43 females) from Kowhai Bush (2008–2015), ordered into multiplexes with their annealing temperature provided in brackets. TG~ markers were developed by Dawson et al. (2010), the Z043B sex marker was developed by Dawson et al. (2016), and Ach~ markers were developed by Preston, Dawson, et al. (2013)

Marker name	Dye	No. alleles	Size range (base pairs)
Multiplex 1 (56 C)			
TG01‐147	HEX	2	278–280
TG04‐004	HEX	2	164–166
TG13‐009	HEX	4	189–199
Z043B (sex marker)	6FAM	2	262–272
Multiplex 2 (60 C)			
Ach006	HEX	5	225–245
Ach007	6FAM	9	232–268
Ach008	HEX	5	264–280
Ach010	6FAM	11	191–217
Ach012	HEX	5	329–356
Ach013	6FAM	7	147–175
Ach014	HEX	5	184–200
Ach020	HEX	3	153–163
Multiplex 3 (60 C)			
Ach001	6FAM	9	189–227
Ach011	6FAM	8	266–280
Ach019	HEX	5	174–184
Ach023	6FAM	12	328–357
Ach030	HEX	10	217–244

**Table A2:** Effect estimates on the log scale from potential predictors of carer provisioning rate in riflemen, modeled as fixed effects in a Poisson‐distributed generalized linear mixed‐effects model (*n* = 1,124 observations). Carer identity (variance component = 0.07), territory (variance component = 0.02) and breeding season (variance component < 0.01) were included as random effects. Second brood and helped are categorical predictors with first broods and unhelped nests as respective reference categories. Brood size, nestling age, date (number of days since 1st September), and time (number of hours since 0700 NZST) were scaled and centered. Carer status, carer sex, and second brood are categorical predictors with breeder, female, and first broods as respective reference categories

Predictor	β ± *SE*	*z*	*p*
*Intercept*	2.37 ± 0.06	37.76	<.001
Proportion of males in brood	−0.12 ± 0.05	−2.42	.016
Brood size	0.27 ± 0.02	17.29	<.001
Nestling age	0.31 ± 0.01	28.24	<.001
Carer status (helper)	−1.01 ± 0.05	−20.40	<.001
Carer sex (male)	0.14 ± 0.05	3.03	.002
Time	−0.03 ± 0.01	−3.69	<.001
Date	−0.09 ± 0.02	−3.44	<.001
Second brood	0.09 ± 0.06	1.45	.146

**Table A3:** Effect estimates on the logit scale from potential predictors of brood sex ratios in riflemen, modeled as fixed effects in a binomially‐distributed generalized linear mixed‐effects model, with the proportion of male offspring in a brood as the response variable (*n* = 93 broods). Breeding season was included as a random effect but explained no variation. Second brood and helped are categorical predictors with first broods and unhelped nests as respective reference categories

Predictor	β ± *SE*	*z*	*p*
*Intercept*	−0.36 ± 0.49	−0.73	.468
Density (no. pairs within 200 m)	0.07 ± 0.05	1.44	.150
Second brood	−0.02 ± 0.36	−0.05	.964
Helped	0.16 ± 0.26	0.62	.533
Brood size	>−0.01	−0.08	.937

**Table A4:** Effect estimates on the logit scale from potential predictors of brood sex ratios in riflemen, modeled as fixed effects in a binomially‐distributed generalized linear mixed‐effects model, with the proportion of male offspring in a brood as the response variable (*n* = 85 broods). Nine unsexed nestlings were assumed to be male. Pair identity (variance component < 0.01) nested within female identity (variance component < 0.01) was included as a random effect along with breeding season (variance component < 0.01). Second brood and helped are categorical predictors with first broods and unhelped nests as respective reference categories

Predictor	β ± *SE*	*z*	*p*
*Intercept*	−0.27 ± 0.52	−0.52	.601
Density (no. pairs within 200 m)	0.10 ± 0.06	1.72	.087
Second brood	−0.06 ± 0.37	−0.17	.864
Helped	0.26 ± 0.27	0.94	.352
Brood size	−0.06 ± 0.13	−0.44	.663
Mother–father relatedness	0.15 ± 0.58	0.26	.795

**Table A5:** Effect estimates on the logit scale from potential predictors of brood sex ratios in riflemen, modeled as fixed effects in a binomially‐distributed generalized linear mixed‐effects model, with the proportion of male offspring in a brood as the response variable (*n* = 85 broods). Nine unsexed nestlings were assumed to be female. Pair identity (variance component < 0.01) nested within female identity (variance component < 0.01) was included as a random effect along with breeding season (variance component < 0.01). Second brood and helped are categorical predictors with first broods and unhelped nests as respective reference categories

Predictor	β ± *SE*	*z*	*p*
*Intercept*	−0.28 ± 0.52	−0.52	.588
Density (no. pairs within 200 m)	0.10 ± 0.06	1.76	.079
Second brood	−0.09 ± 0.37	−0.25	.807
Helped	0.27 ± 0.28	0.97	.331
Brood size	−0.08 ± 0.13	−0.59	.557
Mother–father relatedness	0.19 ± 0.59	0.33	.745

**Table A6:** Effect estimates on the logit scale from potential predictors of brood sex ratios in riflemen, modeled as fixed effects in a binomially‐distributed generalized linear mixed‐effects model, with the proportion of male offspring in a brood as the response variable (*n* = 80 broods). Pair identity nested within female identity, and breeding season, were included as random effects but explained no variation. Second brood is a categorical predictor, with first broods as a reference category

Predictor	β ± *SE*	*z*	*p*
*Intercept*	−0.27 ± 0.53	−0.51	.608
Density (no. pairs within 200 m)	0.10 ± 0.06	1.86	.063
Second brood	−0.18 ± 0.14	−0.28	.779
Number of helpers	0.16 ± 0.26	1.34	.179
Brood size	−0.08 ± 0.14	−0.58	.561
Mother–father relatedness	0.21 ± 0.59	0.36	.721
